# Cerebellar Cognitive Affective Syndrome Presented as Severe Borderline Personality Disorder

**DOI:** 10.1155/2014/894263

**Published:** 2014-03-11

**Authors:** Danilo Pesic, Amir Peljto, Biljana Lukic, Maja Milovanovic, Snezana Svetozarevic, Dusica Lecic Tosevski

**Affiliations:** ^1^Institute of Mental Health, Palmoticeva 37, 11000 Belgrade, Serbia; ^2^Department of Psychology, Faculty of Philosophy, University of Belgrade, Čika Ljubina 18-20, 11000 Belgrade, Serbia; ^3^Belgrade University School of Medicine, Dr. Subotića 8, 11000 Belgrade, Serbia

## Abstract

An increasing number of findings confirm the significance of cerebellum in affecting regulation and early learning. Most consistent findings refer to association of congenital vermis anomalies with deficits in nonmotor functions of cerebellum. In this paper we presented a young woman who was treated since sixteen years of age for polysubstance abuse, affective instability, and self-harming who was later diagnosed with borderline personality disorder. Since the neurological and neuropsychological reports pointed to signs of cerebellar dysfunction and dysexecutive syndrome, we performed magnetic resonance imaging of brain which demonstrated partially developed vermis and rhombencephalosynapsis. These findings match the description of cerebellar cognitive affective syndrome and show an overlap with clinical manifestations of borderline personality disorder.

## 1. Introduction

There are an increasing number of findings supporting the fact that cerebellum, apart from its significance in movement coordination (sensomotoric cerebellum), plays an important role in cognitive and emotional regulation (cognitive and limbic cerebellum) [1]. In comparison with malformations of other parts of cerebellum and the acquired lesions, congenital vermis deficit presents as pronounced slow psychomotor development and worse cognitive functioning (lower IQ) and language skills, as well as affective dysregulation [2]. Rhombencephalosynapsis (RS) is a rare congenital posterior fossa malformation characterised by hypogenesis or agenesis of the vermis, dorsal fusion of cerebellar hemispheres, and fusion of the dentate nuclei and superior cerebellar peduncles [3].

This paper presents a case of a patient diagnosed with borderline personality disorder (BPD). Brain MRI, which was performed because of severe clinical picture and neurological and cognitive deficits, showed partially developed vermis and rhombencephalosynapsis. This sheds a new light on the severity of and nonresponsiveness to medication.

## 2. Case Report

Unemployed, single, 26-year-old woman, with a qualification in catering industry, living with parents, was admitted for treatment to the Institute of Mental Health in Belgrade after one of her multiple suicide attempts by self-poisoning with benzodiazepines. She had been repeatedly hospitalized since she was 16, at first because of polysubstance abuse and later because of self-destructive behaviour, affective instability, and impulsiveness, and had been diagnosed with BPD. Over the past few years psychotropic medications were discontinued. However, she was unable to get a job and establish an emotional relationship. One year prior to admission to the the latest hospitalization, she started abusing benzodiazepines and alcohol again, culminating in a fall and short hospitalization for brain commotion with normal computerised tomography of brain. Afterwards, there were no signs of acute or chronic complications of craniocerebral injury nor emotional and behavioural changes regarding prior state. We acquired data that her mother had a virus infection in the first trimester of pregnancy with scanty hemorrhage. Delivery was vaginal with labour induction, and the baby girl had a pronounced physiological jaundice. Because of hypotonia, rehabilitation was performed. She was able to stand on her own at 15 months and to walk at 18 months and was not manually dexterous. Convergent strabismus was operated when she was two years old. Her attachment pattern was ambivalent. There is a positive heredity on her father's side: one uncle is being treated for depressive disorder and another one for bipolar affective disorder.

Upon admission, the patient showed difficulty in fitting in and it was impossible to establish a therapeutic alliance. She manifested affective instability, was irritable, and verbalised suicidal ideas. During therapeutic weekends she stereotypically inflicted self-harm by cutting her forearm skin and by pressing cigarette butts in her hand dorsum.

Physical examination has not shown any signs of illnesses and common laboratory test values were within reference values. Thyroid function tests were normal. The dominant finding in neurological status was truncal ataxia. Total score on the scale for the assesment and rating of ataxia [4] was 14 out of 40, with dominant findings on stance (5 out of 6), gait (3/8), and sitting (2/4). Convergent strabismus and bilaterally absent corneal reflexes were also registered as well as a small field of temporoparietal left-sided alopecia.

Psychological examination confirmed borderline personality organization, with borderline depression and persistent suicidal risk associated with primary impulse discontrol. Intellectual capacities were at the border of low average: overall IQ was 80, verbal IQ was 84, and manual IQ was 73. The examination emphasized the fact that protrusive negativism was activated regardless of the trigger and that difficulties in attaining aims were possibly also caused by neuropsychologically founded experiential learning capacity reduction.

Various combinations of mood stabilisers and antidepressants together with the second-generation antipsychotics were administered, without mood stabilization and receding of suicidal ideation. A partial response was noted to administration of fluoxetine (40 mg daily) together with olanzapine (15 mg daily). All this led us to a decision to perform neuropsychological examination, electroencephalography (EEG), and MRI.

Neuropsychological examination showed disturbances in complex forms of attention with unfavourable reflection on phonemic fluency and verbal declarative memory, with the presence of retroactive inhibition and dysexecutive syndrome which affect working memory, impede orientation in initial tasks, and reduce benefits from previous experience. A reduction in categorical fluency and dysnomia was evidenced. Constructional praxis was distorted by parietal type; dynamic dyspraxia was evidenced to the left as well as graphesthesia contralaterally (Table 1).

**Figure 1:**
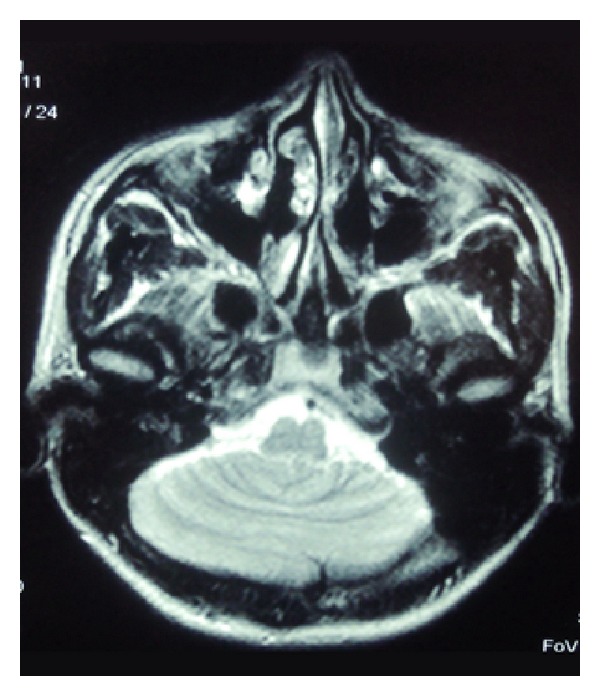
Magnetic resonance imaging of brain which demonstrated partially developed vermis and rhombencephalosynapsis.

The EEG was normal. The MRI has shown partially developed vermis and fusion of cerebellar hemispheres, which was a characteristic of RS (Figure 1). There were no signs of hydrocephalus nor any other central nervous system and extracentral nervous system malformations. The MRI results could explain neurological and neuropsychological findings and presence of “cerebellar cognitive affective syndrome” [5] as well as the persistance of mental health problems.

Taking into consideration the pharmacoresistance and the high sucidal risk, clozapine (150 mg daily), clomipramine (100 mg daily), and lithium carbonate (900 mg daily) were introduced with careful titrating because of potential neurotoxicity and worsening of ataxia. This resulted in receding of suicidal ideation, partial affective stabilization, better impulse control, and abandoning of self-destructive behaviour whereupon the patient was transferred to the out-patient treatment. At the followup her condition is still characterized by affective instability and impulsiveness but without self-harming and suicidal attempts several months later.

## 3. Disscussion

Limbic cerebellum is represented by vermis and fastigial nucleus and cognitive cerebellum by lateral hemispheres of posterior cerebellum [1]. Acquired and congenital lesions of these regions lead to development of nonmotor deficits termed “cerebellar cognitive affective syndrome” (CCAS) [5]. It is characterized by disturbances of executive functions, visual-spatial disorganization, emotional dysregulation (blunting of affect and disinhibited and inadequate behavior), and language deficits (agrammatism and aprosodia) [5].

Impaired social interaction, aggressiveness, pervasive disturbance of behaviour, and self-harming are also more often noted in patients with vermis agenesis [2]. It should be stressed that there are studies which do not show association of acquired lesions of cerebellum with CCAS but only minor cognitive and affective changes. However, they also confirm that CCAS is a consistent finding for congenital malformations [6].

Rhombencephalosynapsis was first described in 1916 after the autopsy of a young man who committed suicide [7]. It can occur as an isolated anomaly or together with different syndromes [3]. Apart from hypotonia, stereotypical head movements, and strabismus, a clinical picture often includes attention disturbance, hyperactivity, and impulsiveness [7]. Verri et al. [8] have described a patient with mild mental retardation, obsessive-compulsive personality disorder, and oral self-mutilations. Nonsyndromic rhombencephalosynapsis can rarely be asymptomatic and associated with normal neuropsychological findings [7].

Our patient manifested trunk ataxia, and in the early development she had hypotonia and developmental dyspraxia. Pronounced affective instability, dysphoria, impulsiveness, and self-harmful behaviour, as well as the dysexecutive syndrome and decrease of working memory together with phonemic fluency reduction, match the description of CCAS as described by Schmahmann [5].

Bilaterally absent corneal reflexes and a small field of temporoparietal left-sided alopecia were also interesting findings, but there were not enough criteria for diagnosing Gomez-Lopez-Hernandez syndrome, which includes rhombencephalosynapsis, trigeminal anesthesia, and bilateral alopecia [9].

Numerous neuropsychiatric manifestations, which can appear together with cerebellar lesions, have been described and can be classified in several domains: attention disturbances, emotional control disturbances, and disturbances which belong to autistic spectrum and psychotic disorders [10]. These disturbances could be explained both by connections with limbic system and prefrontal cortex [1] and the supposed role of cerebellum in social cognition and forming a theory of mind, that is, the ability to attribute mental states to others. Positron emission tomography in healthy volunteers showed a pronounced cerebellar activation while performing tasks which require activation of brain areas in charge of theory of mind [11]. Taking into consideration the fact that the concept of mentalization was formed by applying the concept of the theory of mind in patients with borderline personality disorder, by adding to the objective and cognitive concept of theory of mind a subjective and affective component [12], we could assume that cerebellar dysfunction could play a role in the mentalization problem as well.

On the other hand, fMRI studies showed that processing of negative emotions in patient with BPD was associated with greater activation within insula and posterior cingulate cortex, as well as anterior culmen and posterior declive of the cerebellum, and reduced activation of region that extended from the amygdala to the subgenual anterior cingulate cortex and dorsolateral prefrontal cortex [13]. Two questions remain: whether activation of vermis is primary or compensatory mechanism in BDP patients and how vermal hypoplasia can influence BPD phenotype expression.

We should also take into consideration the possibility that some subradiographic cerebral hemisphere or limbic abnormalities could be present and contribute to the clinical picture in this case.

The presented case shows an overlap between BPD and CCAS and suggests the importance of neurological and neuropsychological evaluation of patients with severe personality disorders.

## Figures and Tables

**Table 1:** Neuropsychological assessment.

Test	Patient	Normative Data	Deviations
WMS-R Verbal memory	7	7 ± 2	/
WMS-R Visual memory	3	7 ± 2	−2.00 SD
WMS-R Attention/Concentration Index	52	>70 sec.	<10 Pr
TMT-A	82	<45 sec.	<10 Pr
TMT-B	197	<98 sec.	<10 Pr
RAVLT *t*	26	55.3 ± 6.6	−4.44 SD
RAVLT *e*	2	14.0 ± 2.0	−6.00 SD
RAVLT *r*	12	14.4 ± 0.8	−3.00 SD
Phonemic fluency tests for divergent thinking	S/8, K/11, L/7	Min. 8	/
Categorical fluency tests for divergent thinking	11	19.58 ± 4.0	−2.15 SD
RCF C	27	35.1 ± 1.5	−5.40 SD
RCF 40′	4.0	22.7 ± 7.0	−2.67 SD
HVOT	21.5/56–60	41–55	Low possibility of impairment
WCST CA	0	5.6 ± 1.0	−5.60 SD
WCST PR	30	13 ± 9.1	+1.86 SD
WCST FMS	0	0.8 ± 1.3	N/A
BNT	50	55.86 ± 2.86	−2.04 SD
BDAE auditory comprehension	11	11.2 ± 1.1	−0.18 SD
BDAE total sentence repetition	LP 7/8	7.7 ± 0.6	−1.17 SD
Ideomotor praxia	8/8	7/8	
Spatial aspects of praxia	10/10	8/10	
Dynamic praxia	D 8	8–10	Left side impairment
	L 5 (3 errors)		
Tactile gnosia	D 3/3	2/3
	L 3/3	
Graphesthesia	D 1/5	4/5	Right side impairment
	L 5/5		
VITI-IQ			
VIQ	84	99.86 ± 14.98	−1.05 SD
PIQ	73	99.37 ± 14.66	−1.80 SD
FSIQ	80	99.19 ± 15.23	−1.26 SD

Wechsler Memory Scale-Revised (WMS-R**)**; Trail Making Test A and B (TMT-A and -B); Rey Auditive Verbal Learning Test (RAVLT): RAVLT *t*: total number of repeated words in five attempts in the RAVLT, RAVLT *e*: number of repeated words after 30 min (evocation) in the RAVLT and RAVLT *r*: number of correctly recognized words; (recognition) in the RAVLT; Rey-Osterrieth Complex Figure Test (RCF): RCF C: copying of the RCF and RCF 40′: 40-minute delayed recall trial; Hooper Visual Organization Test (HVOT); Wisconsin Card Sorting Test (WCST): WCST CA: categories achieved in the WCST, WCST PR: perseverative responses in the WCST and WCST FMS: failures to maintain set in the WCST; Boston Naming Test (BNT); Boston Diagnostic Aphasia Battery (BDAE); Serbian version of Wechsler Adult Intelligence Scale (WAIS)—“Vekslerov Individualni Test Inteligencije” (VITI): verbal IQ (VIQ), performance IQ (PIQ) and full scale IQ (FSIQ).
